# Telomere-Independent Associations of *hTERT* Promoter Polymorphisms with Metabolic Phenotypes and Diabetic Complications

**DOI:** 10.3390/ijms27209292

**Published:** 2026-10-19

**Authors:** Krzysztof Sawicki, Małgorzata Suchecka, Magdalena Matysiak-Kucharek, Marcin Kruszewski, Paulina Wojtyła-Buciora, Piotr Dziemidok, Lucyna Kapka-Skrzypczak

**Affiliations:** 1 Department of Molecular Biology and Translational Research, Institute of Rural Health, 20-090 Lublin, Poland; 2 Department of Diabetes, Institute of Rural Health, 20-090 Lublin, Poland; 3 Centre for Radiobiology and Biological Dosimetry, Institute of Nuclear Chemistry and Technology, 03-195 Warsaw, Poland; 4 Faculty of Medicine and Health Promotion, University of Kalisz, 62-800 Kalisz, Poland; 5 World Institute for Family Health, University of Kalisz, 62-800 Kalisz, Poland

**Keywords:** diabetes, diabetic complication, human telomerase reverse transcriptase, *hTERT* polymorphism

## Abstract

This study investigated association of three *hTERT* promoter polymorphisms (rs2735940, rs7712562, rs2853669) with leukocyte relative telomere length (RTL) and clinical characteristics, in patients with type 1 (T1DM) and type 2 (T2DM) diabetes. Covariate-adjusted ANCOVA revealed no significant direct association between *hTERT* variants and leukocyte RTL in either cohort. However, nominally significant, RTL-independent clinical associations emerged in T2DM patients. Multivariate logistic regression associated the rs2853669 *CC* genotype with lower odds of diabetic neuropathy (*OR* = 0.324, *95% CI*: 0.106–0.989, raw *p* = 0.048). Furthermore, the rs2735940 *TT* genotype was associated with a favorable lipid profile (lower TC, LDL, and non-HDL; raw *p* < 0.05), while rs7712562 *CC* carriers exhibited higher waist circumference (raw *p* = 0.016). While *hTERT* variants were not directly associated with RTL under severe metabolic stress, they exhibited clinically relevant phenotypic associations. Although these exploratory secondary findings did not survive False Discovery Rate (FDR) correction, the nominal association of rs2853669 *CC* with lower neuropathy odds suggests potential non-telomeric pathways associated with mitochondrial protection. These hypothesis-generating observations warrant independent validation in larger, prospective cohorts.

## 1. Introduction

Diabetes is a chronic metabolic disease characterized by hyperglycemia as well as disturbed metabolism of carbohydrates, lipids, and proteins, which generates an enormous health and economic burden worldwide [1,2]. The condition is primarily classified into type 1 diabetes (T1DM), type 2 diabetes (T2DM), gestational diabetes, and unspecified diabetes [2]. T1DM is a chronic autoimmune disease occurring most frequently in children and adolescents (its global incidence is increasing by 2–5% annually). It involves the progressive destruction of pancreatic β-cells, resulting in absolute insulin deficiency [2,3,4]. In contrast, the pathological mechanism of T2DM is characterized by insulin resistance and secondary β-cell dysfunction, which result from the interaction between environmental factors (including epigenetic changes, e.g., methylation) and genetic predispositions. T2DM accounts for approximately 90% of all diagnosed diabetes cases, and its rapid growth means that the number of patients is projected to exceed 500 million by 2030 [1]. At the molecular level, T2DM is a complex multifactorial disorder governed by intricate polygenic networks. Beyond primary metabolic regulators, candidate-gene association studies have highlighted genetic variation across diverse physiological pathways including insulin signaling [5], lipid homeostasis [6], inflammation [7], and cellular stress responses [8,9] as critical determinants of T2DM susceptibility [10].

Inadequate glycemic control in both types of diabetes leads to severe, multiorgan complications [2,4]. Chronic complications of both diseases include microvascular complications (retinopathy, nephropathy, and neuropathy) [2,4,11,12] and macrovascular complications (stroke, acute coronary syndromes, and general cardiovascular diseases) [1,2,11]. Although susceptibility to complications in T2DM has a strong genetic background, most of the responsible *loci* remain undiscovered [11].

Modern medicine extends the average life expectancy of patients with diabetes (especially those with T1DM) and reduces the incidence of complications; however, diseases associated with natural aging may appear earlier in affected individuals [13]. Biological processes, such as persistent oxidative stress and chronic low-grade inflammation, exacerbate cellular senescence and accelerate telomere shortening [1,3,14]. In T1DM patients, shortened telomere length negatively correlates with health profile, constituting an independent factor contributing to higher mortality and progression of nephropathy [3,13]. Interestingly, recent analyses show that telomere shortening is less pronounced in T1DM patients compared to T2DM patients [12]. Understanding molecular mechanisms beyond these differences is crucial for the prevention and treatment of diabetes [2].

Telomeres are nucleoproteins present at the ends of chromosomes (composed of *TTAGGG* repeats) that protect DNA against degradation and chromosomes against fusion, ensuring genomic stability [15,16]. They shorten with each cell division due to incomplete replication, which, along with age, leads to cellular senescence and cell death [17,18]. This process is counteracted by telomerase, the enzyme whose excessive activity provides immortality to cancer cells [16,18]. Excessive telomere shortening, triggered by factors such as aging, is closely linked to the pathogenesis of numerous chronic diseases, including cancer, cardiovascular diseases, pulmonary fibrosis, and diabetes [19].

Telomerase is a ribonucleoprotein enzyme responsible for telomere elongation, composed of an RNA template (TERC) located at chromosome 3q26 and a catalytic reverse transcriptase encoded by the *hTERT* gene located at chromosome 5p15.33, whose activity serves as a rate-limiting factor of the reaction [20,21,22]. Physiologically high telomerase expression occurs in stem and germ cells, whereas it is low in somatic tissues, leading to telomere shortening with age. Its significant reactivation is, in turn, a hallmark of cancer [17,23]. The expression and activity of telomerase depend on polymorphisms in its promoter [17]. Besides protecting genomic stability from replicative damage [24], *hTERT* performs extra-telomeric functions: it modulates gene transcription, responds to oxidative stress, and promotes cell survival and glucose uptake, indicating its potential in mitigating metabolic and diabetic complications [25].

Single-nucleotide polymorphisms (SNPs) within the *hTERT* and *hTERC* genes significantly modify telomerase expression and affect constitutive telomere length [22,26,27,28]. By affecting transcriptional regulation and subsequent telomerase enzymatic activity, these variants can lead to impaired telomere maintenance, telomere dysfunction, disruption of chromosome integrity, and overall genomic instability [10,14,29]. Clinically, this translates to increased mortality and higher susceptibility to age-related diseases, primarily various types of cancer [27,28], but also to the development of T2DM [10,14].

Among the numerous genetic variants of the telomerase complex, three specific SNPs located in the *hTERT* promoter region rs2735940, rs7712562, and rs2853669 have attracted considerable attention. These polymorphisms are known to functionally alter the promoter’s transcriptional activity by modifying binding sites for key transcription factors, thereby modulating baseline telomerase expression [27,30]. Consequently, they have been extensively investigated and linked to altered telomere dynamics, cellular senescence, and susceptibility to various age-related pathologies, primarily diverse malignancies [20,21,31]. More recently, emerging evidence suggests that these specific variants may be associated with metabolic dysregulation, lipid profile alterations, and diabetes susceptibility [25]. However, their precise impact on systemic telomere length and the progression of specific micro- and macrovascular complications in cohorts with both autoimmune (T1DM) and metabolic (T2DM) diabetes remains inadequately explored, providing a strong rationale for their concurrent evaluation.

Diabetes and its complications severely impact patients’ quality of life; therefore, understanding the pathological mechanisms of diabetes associated with biological aging is of key importance for the prevention and treatment of this disease. Given the above, this study aimed to investigate whether selected *hTERT* promoter variants (rs2735940, rs7712562, and rs2853669) are associated with leukocyte relative telomere length (RTL), metabolic parameters, and the occurrence of complications in patients with type 1 and type 2 diabetes.

## 2. Results

### 2.1. Demographic and Clinical Profile of the Study Subjects

The baseline demographic and clinical characteristics of the study cohort are summarized in Table 1. There were no significant differences in sex distribution between the groups (*p* = 0.240). However, patients with T2DM were significantly older than those with T1DM (64.63 vs. 44.34 years, *p* < 0.001), while the T1DM group was characterized by a significantly longer duration of the disease (20.00 vs. 15.00 years, *p* = 0.004). Furthermore, anthropometric assessment revealed that the T2DM patients had a markedly higher body mass index (BMI) (34.15 vs. 25.70 kg/m^2^, *p* < 0.001) and waist circumference (WC) (114.87 vs. 91.40 cm, *p* < 0.001) compared to the T1DM group.

Despite comparable levels of glycaemia, as indicated by HbA1c levels (8.30% in T1DM vs. 8.20% in T2DM, *p* = 0.293), the groups exhibited distinct biochemical profiles. Systemic inflammation, measured by C-reactive protein (CRP), was significantly elevated in the T2DM group (4.57 vs. 1.50 mg/L, *p* < 0.001). Conversely, vitamin D_3_ concentration was higher in patients with T1DM (*p* = 0.007). Lipid profile analysis demonstrated significant differences; the T1DM group had higher levels of total cholesterol, LDL, and HDL (all *p* < 0.001), whereas the level of triglycerides (TG) was elevated in the T2DM group (144.00 vs. 97.00 mg/dL, *p* < 0.001). Non-HDL cholesterol level did not differ significantly between the groups (*p* = 0.244).

The prevalence of diabetic complications varied significantly according to the diabetes type. Patients with T2DM exhibited a significantly higher frequency of diabetic neuropathy (75.2% vs. 36.8%), diabetic foot syndrome (30.1% vs. 12.0%), and diabetic nephropathy (31.5% vs. 8.5%) compared to the T1DM group (all *p* < 0.001). The incidence of diabetic retinopathy was comparable between both groups (31.6% in T1DM vs. 25.9% in T2DM, *p* = 0.307). Notably, crude analysis of relative telomere length (RTL) revealed no significant differences between the unadjusted T1DM and T2DM cohorts (0.99 vs. 1.05, *p* = 0.714), establishing a baseline for further covariate-adjusted models.

### 2.2. Minor Allele Frequencies and Genotype Prevalence of hTERT SNPs

The characteristics of the three investigated *hTERT* single-nucleotide polymorphisms (rs2735940, rs7712562, and rs2853669) are detailed in Table 2. The distributions of genotypes and minor allele frequencies (MAF) for both the T1DM and T2DM cohorts are summarized in Table 3. The genotype distributions for rs2735940 and rs2853669 were consistent with Hardy–Weinberg Equilibrium (HWE) in both patient groups (*p* > 0.05). However, a significant deviation from HWE was observed for the rs7712562 polymorphism in the T1DM cohort (*p* = 0.014), along with a borderline value in the T2DM group (*p* = 0.077, Wigginton exact test). This deviation may be attributed to the relatively small sample size and the particularly low frequency of the rare homozygous *TT* genotype in the studied population.

Analysis of the allele frequency revealed no significant differences between patients with T1DM and T2DM across all three analyzed *loci*, indicating a similar genetic background in both cohorts. The MAF for rs2735940 (*T* allele: 0.474 vs. 0.465, *p* = 0.836), rs7712562 (*T* allele: 0.150 vs. 0.142, *p* = 0.816), and rs2853669 (*C* allele: 0.291 vs. 0.306, *p* = 0.710) were highly comparable. While the overall genotype prevalence for rs2735940 (*p* = 0.964) and rs2853669 (*p* = 0.507) did not differ significantly between the two groups, a notable difference was observed for the rs7712562 variant (*p* = 0.006). Specifically, the rare *TT* homozygous genotype was present in 5.1% of patients with T1DM (*n* = 6), whereas it was absent (0.0%) in the T2DM cohort.

### 2.3. Covariate-Adjusted Relative Telomere Length (log10-RTL) According to hTERT Genotypes

To investigate whether the selected *hTERT* gene polymorphisms are independently associated with telomere attrition in diabetic patients, we compared the leukocyte relative telomere length (log_10_-RTL) across genotypes using an analysis of covariance (ANCOVA). Recognizing the potential impact of clinical variables on telomere biology, the models were adjusted for major confounding factors: age, BMI, and duration of diabetes (Table 4).

Overall, the analysis revealed no statistically significant associations between the evaluated *hTERT* variants and covariate-adjusted leukocyte RTL in either the T1DM or T2DM cohort. For the rs2735940 polymorphism, neither the dominant (*CC* vs. *TT* + *TC*) nor the recessive (*TT* vs. *TC* + *CC*) model exhibited a significant association with leukocyte RTL in patients with T1DM (*p* = 0.258 and *p* = 0.164, respectively) or T2DM (*p* = 0.122 and *p* = 0.594, respectively). Similarly, no significant differences in the adjusted mean leukocyte RTL were observed for the rs2853669 variant across the analyzed genetic models in both study groups (all *p* > 0.05).

Regarding the rs7712562 polymorphism, the dominant model did not reveal any significant differences in leukocyte RTL between the genotype groups in either cohort. Furthermore, the evaluation of the recessive model for this SNP was not feasible (ND) in the T2DM group due to the complete absence of the rare TT homozygous genotype in this population. In the T1DM group, the difference in leukocyte RTL under the recessive model remained statistically non-significant (*p* = 0.179).

Collectively, these findings suggest that, independent of diabetes type, the specific promoter variants of the *hTERT* gene analyzed in this study are not directly associated with systemic leukocyte telomere shortening when properly controlled for major clinical and demographic confounders.

### 2.4. Associations of hTERT Polymorphisms with Clinical and Metabolic Phenotypes

To evaluate the potential associations of the selected *hTERT* variants with clinical characteristics and metabolic profiles, genotype–phenotype association analyses were performed separately for the T1DM and T2DM cohorts. The data are summarized in Table 5.

In the T1DM cohort, no clinical or biochemical parameter, including age, duration of diabetes, BMI, WC, HbA1c, CRP, vitamin D_3_, or lipid profile components (TC, LDL, HDL, non-HDL, and TG), showed statistically significant differences across the genotypes of the analyzed *hTERT* SNPs (all *p* > 0.05).

The T2DM cohort demonstrated distinct association patterns. The rs2735940 polymorphism under a recessive model was significantly associated with key lipid parameters. Patients with the homozygous recessive genotype *TT* exhibited significantly lower levels of TC (125.5 [70.5] vs. 148.0 [59.0] mg/dL, raw *p* = 0.030), LDL cholesterol (57.0 [40.8] vs. 73.5 [41.5] mg/dL, raw *p* = 0.038), and non-HDL cholesterol (94.5 [52.8] vs. 110.0 [54.0] mg/dL, raw *p* = 0.048) compared to the combined heterozygous and dominant homozygous carriers. Furthermore, the rs7712562 polymorphism under a dominant model (*CC* vs. *TT* + *TC*) was significantly associated with WC, with dominant allele carriers presenting with a markedly higher WC (117.0 ± 14.3 vs. 110.0 ± 15.2 cm, raw *p* = 0.016). Other analyzed metabolic and clinical characteristics in the T2DM group showed no significant associations with the *hTERT* gene variants (*p* > 0.05). It should be noted that these specific findings achieved nominal statistical significance (raw *p* < 0.05); however, they did not retain significance following False Discovery Rate (FDR) correction for multiple comparisons across the exploratory secondary outcomes (Supplementary Table S1).

### 2.5. Associations of hTERT Polymorphisms with Diabetic Complications and Sex

To investigate whether *hTERT* gene variants are associated with the risk of diabetic complications, we performed chi-square (*χ^2^*) and Fisher’s exact tests assessing genotype distributions in relation to the prevalence of diabetic neuropathy, diabetic foot syndrome, diabetic retinopathy, and diabetic nephropathy.

In both the T1DM and T2DM cohorts, no statistically significant associations were observed between any of the studied polymorphisms (under additive, dominant, or recessive models) and the occurrence of these four diabetic complications (all *p* > 0.05). Additionally, the distribution of rs2735940, rs7712562, and rs2853669 genotypes did not differ significantly between male and female patients in either diabetes group (all *p* > 0.05), indicating no sex-specific distribution of these genetic variants.

### 2.6. Multivariate Analysis of Diabetic Complications

To evaluate the independent associations of *hTERT* gene polymorphisms with the prevalence of micro- and macrovascular complications, binomial logistic regression models were applied. To avoid model overfitting and ensure stability of the estimates, multivariable models were adjusted for core clinical confounders (age, sex, and duration of diabetes) to calculate the adjusted odds ratios (*OR*) and 95% confidence intervals (*95% CI*) (Table 6).

In the T2DM cohort, multivariable analysis revealed a nominally significant association between the *hTERT* rs2853669 variant and diabetic neuropathy. Under the recessive model, the homozygous *CC* genotype was associated with lower odds of diabetic neuropathy compared to the combined *TC* and *TT* genotypes (*OR* = 0.324, *95% CI*: 0.106–0.989, raw *p* = 0.048; Table 6). None of the included clinical covariates showed a significant independent association with neuropathy within this specific model (*p* > 0.05). While this inverse association was nominally significant in the initial multivariable model, it did not withstand rigorous False Discovery Rate (FDR) adjustment for multiple testing across all exploratory secondary outcomes (Supplementary Table S1). No other statistically significant relationships were identified between the studied *hTERT* SNPs and other diabetic complications (retinopathy, nephropathy, or diabetic foot syndrome) in the T2DM group.

In the T1DM cohort, multivariable logistic regression did not reveal any statistically significant associations between the analyzed *hTERT* polymorphisms (rs2735940, rs7712562, and rs2853669) and the occurrence of any evaluated diabetic complications (all *p* > 0.05).

## 3. Discussion

This study investigated a potential association of three promoter polymorphisms of the *hTERT* gene, namely rs2735940, rs7712562, and rs2853669, with leukocyte RTL, metabolic characteristics, and chronic micro- and macrovascular complications in separate cohorts of patients with T1DM (*n* = 117) and T2DM (*n* = 144) diabetes mellitus. To the best of our knowledge, this is the first study directly comparing these specific genetic variants and adjusted RTL dynamics side-by-side in distinct autoimmune and metabolic diabetic landscapes.

Our most promising secondary finding is that the homozygous *CC* genotype of the *hTERT* rs2853669 variant demonstrated a nominal inverse association with the odds of diabetic neuropathy in patients with T2DM (*OR* = 0.324, *95% CI*: 0.106–0.989, raw *p* = 0.048). The rs2853669 polymorphism is located within the *hTERT* promoter region and documented to disrupt the consensus binding sequences for ETS (E26 transformation-specific) and TCF (T-cell factor) transcription factors, which act as positive regulators of *hTERT* gene transcription. Disruption of these core motifs typically leads to compromised transcriptional efficiency, reduced gene expression, and lower baseline telomerase activity [27,30,32].

The rs2853669 polymorphism has been extensively characterized as a molecular driver of tumor aggressiveness and clinical prognosis in various oncological settings, such as bladder cancer [33], thyroid carcinoma [15], oral leukoplakia [34], and acute myeloid leukemia [35,36]; however, its relevance in non-oncological, degenerative pathologies is increasingly recognized. Notably, in sarcoidosis, the rs2853669 *CC* genotype has been shown to predict progressive loss of respiratory lung function [37], illustrating its systemic role in modulating tissue resilience and chronic organ degradation.

To explain the potential implications of the *CC* genotype in our T2DM cohort under chronic metabolic stress, one must look beyond classical telomere maintenance to the non-canonical functions of *hTERT*. Under conditions of systemic low-grade chronic inflammation and high oxidative stress, *hTERT* is actively exported from the nucleus into the mitochondria [11]. Within the mitochondrial matrix, *hTERT* acts as a direct shield for mitochondrial DNA (*mtDNA*) and transfer RNAs, preserving *mtDNA* integrity against oxidative insults, enhancing mitochondrial membrane potential, and reducing endogenous reactive oxygen species (ROS) generation, thereby inhibiting apoptosis [38]. The literature indicates that neurons and Schwann cells are particularly susceptible to glucose-induced mitochondrial dysfunction and oxidative injury [39].

Furthermore, functional studies have revealed that rs2853669 *CC* homozygotes often co-occur with lower fasting plasma glucose levels, possibly because *hTERT* interacts with metabolic pathways regulating cellular glucose uptake and the pentose phosphate pathway [25]. Peripheral glucose homeostasis relies on precise activation of the insulin receptor and downstream IRS-1/PI3K/Akt signaling cascade. As documented by de Lorenzo et al. (2013) [40], inherited polymorphisms in genes encoding endogenous negative regulators and inhibitors of insulin signaling (such as *ENPP1*, *PTPN1*, and *TRIB3*) impair post-receptor signal transduction, thereby blunting insulin-stimulated GLUT4 translocation and peripheral glucose transport and promoting T2DM susceptibility and metabolic dysregulation. Consequently, any non-canonical modulation of cellular metabolic signaling mediated by *hTERT* promoter variants likely operates within a broader polygenic background. An integrated perspective suggests that genetic variation in core insulin-signaling inhibitors and extra-telomeric regulators, like *hTERT*, cooperatively modulates an individual’s genotype-dependent metabolic susceptibility and overall glycemic control.

The proposed extratelomeric, mitochondrial-protective functions of *hTERT* should also be considered within the broader context of genetic susceptibility to metabolic stress. Oxidative damage to mitochondria is a major driver of insulin resistance and T2DM pathogenesis. Moreover, genetic variations in endogenous antioxidant-defense pathways heavily modulate individual susceptibility to oxidative injury. For example, metallothioneins (MTs) are critical for scavenging free radicals, sensing cellular redox status, and controlling mitochondrial zinc delivery. Polymorphisms in metallothionein-encoding genes, such as the *MT1A* rs11640851 variant, significantly alter the risk of T2DM development by affecting intracellular antioxidant capacity [41]. Acknowledging this broader genetic landscape of endogenous antioxidant defense provides essential context for our findings, suggesting that the potential association of *hTERT* variants with oxidative injury and diabetic neuropathy likely operates within a larger, polygenic network of cellular stress responses.

Consequently, we hypothesize that the *hTERT* rs2853669 *CC* genotype may promote a metabolic phenotype characterized by reduced local glucotoxicity and enhanced mitochondrial resilience in peripheral nerves, synergistically shielding them from apoptotic degeneration and subsequent neuropathic damage. However it must be emphasized that this secondary finding exhibited borderline statistical significance (raw *p* = 0.048) and did not withstand FDR correction for multiple comparisons (Supplementary Table S1). Consequently, this association should be strictly interpreted as an exploratory, hypothesis-generating observation that necessitates independent validation in larger, prospective cohorts.

In contrast to the neuropathic association, our covariate-adjusted ANCOVA models did not reveal any statistically significant direct associations between the analyzed *hTERT* polymorphisms and leukocyte RTL in either T1DM or T2DM patients. On a molecular level, the promoter variant rs2735940 has been shown to significantly modify transcriptional activity, with the *T* allele exhibiting approximately 25% higher efficiency compared to the *C* allele [20,21]. Consequently, the rs2735940 *CC* genotype has been linked to accelerated telomere shortening [20,42]. However, the lack of genetic association with RTL revealed by this study partially diverges from some other observations in the Polish population. Specifically, Gutmajster et al. (2018) [25], in their analysis of elderly obese Polish subjects from the PolSenior cohort, reported that rs2853669 *CC* homozygotes exhibited the shortest RTL, and the difference reached statistical significance exclusively in their T2DM group. Conversely, in our covariate-adjusted ANCOVA models, we found no significant differences in adjusted log_10_-RTL across the rs2853669 genotypes in T2DM patients (*p* = 0.883).

This discrepancy can be logically reconciled by differences in cohort composition and statistical design. While the PolSenior cohort studied by Gutmajster et al. (2018) [25] targeted an elderly, obese population, where long-term environmental effects on telomeres were maximally accumulated, our T2DM cohort was relatively younger (64.63 ± 9.87 vs. 76.9 ± 7.7 years). Furthermore, our use of ANCOVA models adjusted simultaneously for key clinical confounders including age, BMI, and diabetes duration likely controlled for a massive portion of the variance in leukocyte telomere length, thereby “washing out” the subtle, baseline genetic influence of the promoter SNPs.

In line with our assumption, Gutmajster et al. (2018) [25] also noted that overall RTL did not differ significantly between T2DM patients and controls, suggesting that under the extreme and chronic physiological stress of diabetes mellitus, environmental and clinical factors exert a far more dominant influence on systemic telomere attrition than subtle promoter-driven genetic variations. Chronically elevated systemic inflammation and glucotoxicity drive significant oxidative telomeric DNA damage [23,43], while the severe metabolic pressure of diabetes potentially masks or overrides the subtle influence of baseline genetic variations on telomere length [23].

Systemic inflammation in T2DM is not merely a secondary consequence of metabolic dysfunction but is heavily modulated by genetic variations in innate inflammatory and intracellular signaling pathways. For instance, inherited polymorphisms in key immune-related genes, such as *TNFα*, *IL6*, and *CRP*, have been shown to significantly influence metabolic phenotypes, including fasting insulin level, lipid dysregulation (e.g., reduced HDL), and circulating concentration of acute-phase proteins. Genetically controlled elevation of these pro-inflammatory cytokines establishes a hostile systemic environment that exacerbates oxidative stress and accelerates telomere shortening. The synergistic interaction between genetic susceptibility to heightened inflammatory response and compromised telomerase activity may collectively drive a profound dysregulation of glucose and lipid metabolism, ultimately accelerating the progression of diabetic microvascular complications [44].

Nevertheless, we uncovered several intriguing, genotype-specific associations with baseline metabolic phenotypes that appear to function independently of systemic leukocyte RTL, and therefore warrant discussion. In the T2DM group, the recessive *TT* genotype of the rs2735940 variant was significantly associated with a more favorable lipid profile, characterized by lower total cholesterol, LDL cholesterol, and non-HDL cholesterol compared to *TC* and *CC* carriers. Given that the rs2735940 *TT* genotype exhibits higher promoter activity [25], this genotype may enhance *hTERT’s* extra-nuclear roles in lipid metabolism, potentially by interacting with the Wnt/β -catenin signaling pathway, which is a major regulator of adipogenesis and lipid homeostasis [45,46]. Furthermore, rs2735940 has been previously associated with susceptibility to peripheral artery disease [42], demonstrating its clinical relevance in vascular pathology.

The observed association between the *hTERT* rs2735940 polymorphism and a more favorable lipid profile should be contextualized within a broader landscape of genetically regulated lipid handling in T2DM. Diabetic dyslipidemia is a complex, multifactorial trait driven by insulin resistance and genetic susceptibility affecting lipoprotein assembly, intravascular processing, and clearance. Evidence from candidate-gene evaluations in diabetic cohorts, such as those by Tetik et al. (2017) [47], demonstrates that polymorphisms in key enzymes, receptors, and regulatory proteins governing lipoprotein metabolism (including *LPL*, *LIPC*, *SCARB1*, *LCAT*, *CETP*, and adipokines, like *ADIPOQ* and *RETN*) significantly modulate plasma lipid traits, insulin sensitivity, and the risk of diabetic dyslipidemia. Furthermore, cellular efflux mechanisms, particularly those mediated by ATP-binding cassette transporter A1 (ABCA1), critically regulate high-density lipoprotein (HDL) maturation and reverse cholesterol transport, with *ABCA1* genetic variants directly modulating HDL concentrations and susceptibility to diabetic complications [48]. Within this polygenic network, where multiple enzyme-coding genes and transporters cooperatively regulate lipid homeostasis, the *hTERT* rs2735940 variant likely does not act in isolation. Instead, telomere-related genetic variation may intersect with these established enzymatic and transport pathways of lipid clearance and reverse cholesterol transport, collectively shaping the overall metabolic phenotype and microvascular risk profile in diabetic patients.

Additionally, we found that rs7712562 dominant allele carriers (*CC*) had a significantly higher waist circumference compared to those with the *TT* + *TC* genotype. The rs7712562 polymorphism, extending from exon 2 to the near-promoter region of *hTERT*, is a documented locus of clinical significance previously linked to tumor susceptibility, most notably prostate cancer risk [31]. Our findings reinforce the biological link between *hTERT* genetic variation and abdominal adiposity, a core feature of metabolic syndrome and insulin resistance in T2DM.

The present study has several notable strengths. To the best of our knowledge, this is the first genetic study evaluating these specific *hTERT* variants and leukocyte RTL in a cohort of Polish patients, comparing both major types of diabetes (T1DM and T2DM) side-by-side. However, some limitations must be acknowledged. It is crucial to recognize that genetic association studies involving candidate SNP frequently exhibit population-dependent effects. As demonstrated by comprehensive meta-analyses in T2DM genetics, such as the evaluation of *RETN* gene polymorphisms, individual SNP associations can vary significantly across different ethnic groups, and studies among different ethnicities frequently reveal inconsistencies. Such conflicting findings often arise due to incomplete study designs or low statistical significance caused by small sample sizes [49]. Therefore, the genotypic associations observed in our specific cohort may not be universally applicable. Above mentioned meta-analytic evidence highlights the absolute necessity for independent validation of our *hTERT* findings across diverse populations and larger cohorts to confirm their broader clinical relevance.

The cross-sectional design of our study prevents us from establishing direct causal relationships over time. While our sample sizes (T1DM *n* = 117 and T2DM *n* = 144) were sufficient to detect major associations, such as the rs2853669 association with neuropathy, they may not be large enough to detect very subtle genetic influences on RTL or other low-prevalence complications. Notably, the rs7712562 polymorphism exhibited a significant deviation from Hardy–Weinberg Equilibrium (HWE) specifically within the T1DM cohort (*p* = 0.014). While this may primarily reflect a modest sample size and rarity of the mutant *TT* genotype in this specific sub-cohort, other potential contributing factors must be acknowledged. First, our hospital-based recruitment may introduce a specific sampling characteristics that does not perfectly reflect random distribution of the general population. Specifically, the recruitment of a consecutive, unselected real-world clinical cohort, while highly representative of everyday hospital practice, inherently lacked strict exclusion criteria for potential unmeasured confounders, such as subclinical infections or asymptomatic comorbidities. Second, although the Polish population is generally considered genetically homogeneous, subtle population structure or stratification cannot be entirely ruled out. Finally, regarding genotyping uncertainty, the TaqMan^®^ methodology is generally considered as highly reliable, while to successfully exclude sample contamination we routinely utilized non-template (negative) controls in every qPCR run. However, because extensive independent re-genotyping of duplicates was not performed as part of the study protocol, a minor degree of genotyping uncertainty, such as potential allele dropout, cannot be definitively excluded as a factor contributing to the observed HWE deviation.

Another notable limitation is the profound baseline clinical discrepancy between the T1DM and T2DM cohorts, particularly regarding age and adiposity metrics (BMI and waist circumference). While our multivariable and ANCOVA models were rigorously adjusted for these major confounders to isolate the independent effects of genetic variants, these massive baseline disparities reflect the inherently distinct pathophysiological trajectories of autoimmune versus metabolic diabetes. Consequently, these fundamental biological differences complicate direct head-to-head comparisons between the two groups. Therefore, our findings should be interpreted within the specific clinical context of each diabetes type rather than as directly equivalent biological phenomena.

Next, a major limitation of our study is that relative telomere length (RTL) was measured exclusively in circulating peripheral blood leukocytes. While leukocyte RTL is a widely utilized systemic biomarker of biological aging, leukocytes may serve as poor proxies for telomere dynamics and local *hTERT* expression in specific target tissues heavily affected by diabetic complications, such as peripheral nerve terminals. Consequently, the lack of significant association between *hTERT* polymorphisms and leukocyte RTL does not completely rule out the existence of an alternate, tissue-specific mechanism. It is plausible that non-telomeric functions of *hTERT*, such as local mitochondrial protection and reduction of oxidative stress, operate directly within the nervous system or other disease target tissues, independent of systemic leukocyte telomere length.

Finally, given the large number of exploratory secondary comparisons (*m* = 261) performed to assess various clinical and metabolic parameters, we applied the Benjamini–Hochberg False Discovery Rate (FDR) adjustment (Supplementary Table S1). As is heavily anticipated in modestly sized candidate-gene studies, the nominally significant associations (such as with diabetic neuropathy and specific lipid profiles) did not survive this rigorous multiple-testing penalty. Consequently, these specific genotype–phenotype correlations must be interpreted strictly as exploratory, hypothesis-generating observations that highlight potential extratelomeric mechanisms, but absolutely necessitate independent prospective validation in substantially larger multicenter cohorts.

## 4. Materials and Methods

### 4.1. Study Population and Inclusion/Exclusion Criteria

The study cohort consisted of 117 T1DM and 144 T2DM patients (131 women and 130 men), aged between 18 and 87 years, who volunteered to participate in the study during their in-patient stay at the Department of Diabetes at the Institute of Rural Health in Lublin, Poland. The study was conducted between 2025 and 2026. Written informed consent was obtained from each study recruit. The study protocol was approved by the local Bioethics Committee of the Institute of Rural Health in Lublin (approval no. 7/2025/IMW).

To minimize population stratification bias inherent to genetic association studies, the cohort was restricted to individuals of Caucasian descent and Polish nationality. Patients were recruited as a consecutive, real-world clinical cohort. The inclusion criteria comprised: (1) clinically confirmed diagnosis of T1DM or T2DM; (2) age of 18 years or older; and (3) Caucasian race/Polish nationality. To accurately reflect an unselected hospital population, strict exclusion criteria were kept to a minimum. Patients were excluded only if they met any of the following criteria: (1) pregnancy; (2) lack of conscious written informed consent; or (3) missing essential clinical, biochemical, or genotypic data necessary for the final analysis.

### 4.2. Data Collection and Biochemical Analysis

To obtain a comprehensive assessment of the patients’ clinical status, demographic and medical data were extracted from hospital records. These data encompassed information obtained during standardized clinical interviews, anthropometric measurements (e.g., body mass index, waist circumference), and the results of routine biochemical tests (including HbA1c, lipid profiles, and C-reactive protein) conducted upon admission to the hospital, as previously described in detail by Sawicki et al. (2024) [50]. Furthermore, the definitive presence or absence of specific chronic micro- and macrovascular complications (diabetic neuropathy, nephropathy, retinopathy, and diabetic foot syndrome) was recorded for each patient based on formal clinical evaluations.

### 4.3. Blood Sample Collection and DNA Extraction

Peripheral venous blood samples were collected from each patient into 4.9 mL tubes containing K_3_EDTA (Sarstedt, Nümbrecht, Germany), aliquoted, and stored at −20 °C. Genomic DNA was extracted from 200 µL of whole blood using the QIAamp^®^ DNA Mini Kit (Qiagen, Hilden, Germany) in strict accordance with the manufacturer’s protocol. The concentration and purity of the isolated DNA were accurately assessed using a NanoDrop^®^ 1000 spectrophotometer (Thermo Fisher Scientific, Waltham, MA, USA). The extracted DNA was subsequently aliquoted and stored at −20 °C until further genetic analyses.

### 4.4. Leukocyte Telomere Length Measurement

Leukocyte relative telomere length (RTL) was determined from the extracted genomic DNA by quantitative real-time PCR (qPCR) using an ABI 7500 Fast Real-Time PCR System (Applied Biosystems^®^, Foster City, CA, USA). The results were calculated as the telomere repeat copy number to single-copy gene (*36B4*) amplification ratio (T/S ratio). The detailed laboratory protocol, including reaction mixture composition, primer sequences, and thermal cycling conditions, was performed exactly as previously described by Sawicki et al. (2024) [50]. All samples were amplified in triplicate to ensure technical reproducibility. For subsequent parametric statistical analyses, the raw T/S ratios were log_10_-transformed (log_10_-RTL) to approximate a normal data distribution.

### 4.5. Determination of hTERT Single-Nucleotide Polymorphisms

Genotyping was performed on an ABI 7500 Fast Real-Time PCR System (Applied Biosystems, Foster City, CA, USA) using pre-designed TaqMan^®^ SNP Genotyping Assays (Thermo Fisher Scientific, Waltham, MA, USA) according to the manufacturer’s protocol. The two-step reaction protocol consisted of PCR amplification followed by endpoint fluorescence detection. Each reaction mixture had a total volume of 25 µL and contained 2.5 µL of genomic DNA (30 ng/µL), 12.5 µL of 2X TaqMan^®^ Genotyping Master Mix, 1.25 µL of 20X TaqMan^®^ SNP Genotyping Assay (for rs2735940, rs7712562, or rs2853669), and 8.75 µL of nuclease-free water (Invitrogen, Carlsbad, CA, USA). The thermal cycling conditions comprised a pre-PCR read at 60 °C for 1 min and an initial denaturation stage at 95 °C for 10 min, followed by 40 cycles of denaturation at 95 °C for 15 s and annealing/extension at 60 °C for 1 min, with a final post-PCR read at 60 °C for 1 min. To monitor potential sample contamination and ensure assay reliability, non-template (negative) controls were routinely included in every amplification run. All alleles were normalized and reported according to the forward strand of the human reference genome (GRCh38/hg38).

### 4.6. Statistical Analysis

Statistical analysis was performed using MS Office Excel 2013 (Microsoft Corporation, Redmond, WA, USA), GraphPad Prism version 9.5.1 (GraphPad Software Inc., San Diego, CA, USA), and the jamovi project (version 2.6, 2025).

Leukocyte RTL was designated as the primary outcome of the study, whereas clinical complications, anthropometric measurements, and metabolic parameters were strictly classified as exploratory secondary outcomes.

Continuous variables were assessed for normality using the Shapiro–Wilk test. Due to its right-skewed distribution, RTL was log_10_-transformed before analysis to meet the assumptions of parametric tests. Hardy–Weinberg Equilibrium (HWE) was verified using the chi-square and Wigginton’s exact tests. Quantitative clinical parameters were compared between genotypes or diabetes types (T1DM vs. T2DM) using a *t*-test for independent samples or Mann–Whitney U test, depending on data distribution. To compare log_10_-RTL between groups while controlling for confounding factors, an analysis of covariance (ANCOVA) was applied with age, BMI, and duration of diabetes as covariates. Categorical variables were evaluated using the chi-square test or Fisher’s exact test. Multivariable binomial logistic regression analysis, adjusted for relevant clinical covariates (including age, sex, and disease duration), was performed to calculate odds ratios (*ORs*) and 95% confidence intervals (*CIs*) for diabetes complications.

Nominal statistical significance was initially defined as a two-tailed *p* < 0.05. However, to rigorously address the multiple testing burden associated with the exploratory secondary analyses, the Benjamini–Hochberg False Discovery Rate (FDR) adjustment was systematically applied to these evaluations (*m* = 261 tests).

Genetic models were defined as follows: for a given SNP with a major allele (*A*) and a minor allele (*a*), the dominant model was coded as grouping heterozygous and homozygous minor allele carriers against homozygous major allele carriers (*Aa* + *aa* vs. *AA*), while the recessive model grouped homozygous minor allele carriers against the combined heterozygous and homozygous major allele carriers (*aa* vs. *AA* + *Aa*).

## 5. Conclusions

In conclusion, our findings demonstrate that while *hTERT* promoter polymorphisms are not directly associated with leukocyte telomere length under the heavy metabolic pressure of diabetes, they may exhibit clinically relevant phenotypic associations independent of systemic leukocyte RTL. Specifically, the *hTERT* rs2853669 *CC* genotype demonstrated a nominal association with lower odds of diabetic neuropathy in T2DM. Although this exploratory finding did not survive rigorous False Discovery Rate correction, it may serve as a robust hypothesis-generating observation pointing towards potential extratelomeric, mitochondrial-protective pathways. Future longitudinal studies and functional in vitro analyses are needed to fully elucidate the exact molecular mechanisms by which these promoter variants interact with metabolic and neuropathic pathways.

## Figures and Tables

**Table 1:** Comparison of baseline demographic and clinical characteristics between T1DM and T2DM cohorts.

Parameter	T1DM (*n* = 117)	T2DM (*n* = 144)	*p*-Value
**Demographics & Anthropometrics**			
Female sex, *n* (%)	63 (53.8)	67 (46.5)	0.240 ^c^
Age (years), mean ± SD	44.34 ± 16.65	64.63 ± 9.87	**<0.001 ^a^**
Duration of diabetes (years), median [IQR]	20.00 [18.00]	15.00 [10.00]	**0.004 ^b^**
BMI (kg/m^2^), median [IQR]	25.70 [6.45]	34.15 [9.43]	**<0.001 ^b^**
WC (cm), mean ± SD	91.40 ± 14.92	114.87 ± 14.83	**<0.001 ^a^**
**Biochemical Markers**			
RTL, median [IQR]	0.99 [0.68]	1.05 [0.44]	0.714 ^b^
HbA1c (%), median [IQR]	8.30 [2.40]	8.20 [2.38]	0.293 ^b^
CRP (mg/L), median [IQR]	1.50 [3.81]	4.57 [10.37]	**<0.001 ^b^**
Vitamin D_3_ (ng/mL), median [IQR]	23.90 [17.50]	19.60 [14.95]	**0.007 ^b^**
TC (mg/dL), median [IQR]	169.00 [59.00]	145.00 [58.00]	**<0.001 ^b^**
LDL (mg/dL), median [IQR]	94.00 [47.00]	70.50 [45.25]	**<0.001 ^b^**
HDL (mg/dL), median [IQR]	51.50 [19.25]	36.00 [14.50]	**<0.001 ^b^**
Non-HDL (mg/dL), median [IQR]	118.00 [53.00]	107.00 [51.00]	0.244 ^b^
TG (mg/dL), median [IQR]	97.00 [53.00]	144.00 [50.00]	**<0.001 ^b^**
**Diabetic Complications**			
Diabetic neuropathy, *n* (%)	43 (36.8)	106 (75.2)	**<0.001 ^c^**
Diabetic foot syndrome, *n* (%)	14 (12.0)	43 (30.1)	**<0.001 ^c^**
Diabetic retinopathy, *n* (%)	37 (31.6)	37 (25.9)	0.307 ^c^
Diabetic nephropathy, *n* (%)	10 (8.5)	45 (31.5)	**<0.001 ^c^**

Data are expressed as a mean ± standard deviation (SD), median [interquartile range, IQR], or number of cases (percentage). Statistically significant values (*p* < 0.05) are indicated in bold. Statistical differences between groups were evaluated using: ^a^ Student’s *t*-test for independent samples; ^b^ Mann–Whitney U test; ^c^ Chi-square test. T1DM, type 1 diabetes mellitus; T2DM, type 2 diabetes mellitus; BMI, body mass index; WC, waist circumference; RTL, relative telomere length; HbA1c, glycated hemoglobin; CRP, C-reactive protein; TC, total cholesterol; LDL, low-density lipoprotein cholesterol; HDL, high-density lipoprotein cholesterol; TG, triglycerides.

**Table 2:** Characteristics of the investigated *hTERT* single-nucleotide polymorphisms.

Polymorphism (dbSNP)	Genomic Position (GRCh38)	Minor Allele	European MAF
rs2735940	Chr5: 1,296,371	*C*	0.486
rs7712562	Chr5: 1,295,957	*T*	0.152
rs2853669	Chr5: 1,295,234	*C*	0.310

dbSNP, Single Nucleotide Polymorphism Database reference number; GRCh38, Genome Reference Consortium Human Build 38; MAF, minor allele frequency based on European populations.

**Table 3:** Minor allele frequencies and genotype prevalence of *hTERT* single-nucleotide polymorphisms in T1DM and T2DM patients.

Polymorphism/Parameter	T1DM (*n* = 117)	T2DM (*n* = 144)	OR (95% Cl)	*p*-Value
**rs2735940 (** ***T/C*** **)**				
Genotype, *n* (%)				0.964 ^a^
*TT*	26 (22.2)	30 (20.8)		
*TC*	59 (50.4)	74 (51.4)		
*CC*	32 (27.4)	40 (27.8)		
MAF (*T* allele)	0.474	0.465	0.964 (0.682–1.360)	0.836 ^a^
HWE (*p*-value)	0.904	0.694		
**rs7712562 (** ***T/C*** **)**				
Genotype, *n* (%)				**0.006 ^b^**
*TT*	6 (5.1)	0 (0.0)		
*TC*	23 (19.7)	41 (28.5)		
*CC*	88 (75.2)	103 (71.5)		
MAF (*T* allele)	0.150	0.142	0.944 (0.579–1.540)	0.816 ^a^
HWE (*p*-value)	**0.014**	0.077 ^c^		
**rs2853669 (** ***T/C*** **)**				
Genotype, *n* (%)				0.507 ^a^
*TT*	59 (50.4)	74 (51.4)		
*TC*	48 (41.0)	52 (36.1)		
*CC*	10 (8.6)	18 (12.5)		
MAF (*C* allele)	0.291	0.306	0.931 (0.638–1.360)	0.710 ^a^
HWE (*p*-value)	0.957	0.074		

Data are expressed as number of cases (percentage) or as frequencies. Statistically significant values (*p* < 0.05) are indicated in bold. Statistical differences were evaluated using: ^a^ Pearson’s Chi-square test; ^b^ Fisher’s exact test (used due to low expected counts in the TT genotype subgroup). ^c^ HWE *p*-value was calculated using the Wigginton exact test due to low minor homozygous genotype frequency. T1DM, type 1 diabetes mellitus; T2DM, type 2 diabetes mellitus; MAF, minor allele frequency; OR, odds ratio; 95% CI, 95% confidence interval; HWE, Hardy–Weinberg Equilibrium.

**Table 4:** Covariate-adjusted leukocyte relative telomere length (log_10_-RTL) according to *hTERT* genetic models.

Polymorphism/Genetic Model	Genotype Comparison	T1DM (*n* = 117)		T2DM (*n* = 144)	
		Adjusted Mean ± SE	*p*-Value	Adjusted Mean ± SE	*p*-Value
rs2735940					
Dominant	*CC* vs. *TT + TC*	−0.059 ± 0.039 vs. −0.006 ± 0.023	0.258	−0.049 ± 0.026 vs. −0.001 ± 0.016	0.122
Recessive	*TT* vs. *TC + CC*	0.034 ± 0.044 vs. −0.036 ± 0.023	0.164	−0.001 ± 0.029 vs. −0.018 ± 0.015	0.594
rs7712562					
Dominant	*CC* vs. *TT + TC*	−0.033 ± 0.023 vs. 0.020 ± 0.041	0.262	−0.022 ± 0.016 vs. 0.004 ± 0.026	0.393
Recessive	*TT* vs. *TC + CC*	−0.137 ± 0.088 vs. −0.014 ± 0.020	0.179	ND	ND
rs2853669					
Dominant	*TT* vs. *CC + TC*	−0.029 ± 0.029 vs. −0.012 ± 0.028	0.675	−0.018 ± 0.019 vs. −0.010 ± 0.019	0.774
Recessive	*CC* vs. *TC + TT*	0.052 ± 0.069 vs. −0.027 ± 0.021	0.269	−0.009 ± 0.037 vs. −0.015 ± 0.014	0.883

Data are presented as Estimated Marginal Means ± Standard Error (SE) derived from Analysis of Covariance (ANCOVA) models. All models were adjusted for baseline confounding factors: age, BMI, and duration of diabetes. T1DM, type 1 diabetes mellitus; T2DM, type 2 diabetes mellitus; ND, no data (evaluation not feasible due to the complete absence of the rare homozygous genotype in this specific cohort).

**Table 5:** Nominally significant associations between *hTERT* gene polymorphisms and clinical/metabolic parameters in the T2DM cohort.

Clinical/Metabolic Parameter	Genotype 1	Genotype 2	Raw *p*-Value
rs2735940 (Recessive model)	*TT*	*TC + CC*	
TC (mg/dL)	125.5 [70.5]	148.0 [59.0]	**0.030 ^a^**
LDL (mg/dL)	57.0 [40.8]	73.5 [41.5]	**0.038 ^a^**
Non-HDL (mg/dL)	94.5 [52.8]	110.0 [54.0]	**0.048 ^a^**
rs7712562 (Dominant model)	*CC*	*TT + TC*	
WC (cm)	117.0 ± 14.3	110.0 ± 15.2	**0.016 ^b^**

Data are expressed as a median [interquartile range, IQR] or a mean ± standard deviation (SD). Only nominally significant associations (raw *p* < 0.05) are presented and indicated in bold. All other analyzed clinical and biochemical parameters, as well as the prevalence of specific diabetic complications and sex distribution, showed no significant differences across genotypes (*p* > 0.05). Importantly, these findings did not retain statistical significance following False Discovery Rate (FDR) adjustment for multiple comparisons (Supplementary Table S1). Statistical differences between genotype groups were evaluated using: ^a^ Mann–Whitney U test; ^b^ Student’s *t*-test for independent samples. T2DM, type 2 diabetes mellitus; TC, total cholesterol; LDL, low-density lipoprotein cholesterol; Non-HD, non-high-density lipoprotein cholesterol; WC, waist circumference.

**Table 6:** Multivariable binomial logistic regression analysis for diabetic neuropathy in the T2DM cohort.

Predictor	Adjusted OR	95% CI	Raw *p*-Value
*hTERT* rs2853669 (Recessive: *CC* vs. *TC + TT*)	0.324	0.106–0.989	**0.048**
Age (years)	1.037	0.988–1.087	0.139
Sex (Male vs. Female)	1.485	0.615–3.590	0.380
Duration of diabetes (years)	1.033	0.987–1.091	0.242

Statistically significant values (raw *p* < 0.05) are indicated in bold. The multivariable model was evaluated using binomial logistic regression in the T2DM cohort with diabetic neuropathy as the dependent variable, simultaneously adjusted for the genetic predictor and core clinical covariates (age, sex, and duration of diabetes). While the genetic association with neuropathy was nominally significant in this specific model, it did not retain formal statistical significance following rigorous False Discovery Rate (FDR) adjustment for multiple testing (Supplementary Table S1). T2DM, type 2 diabetes mellitus; OR, odds ratio; 95% CI, 95% confidence interval.

## Data Availability

The datasets used and/or analyzed during the current study are available from the corresponding authors on reasonable request.

## Supplementary Materials

The following supporting information can be downloaded at: https://www.mdpi.com/article/10.3390/ijms27209292/s1.

## Abbreviations

The following abbreviations are used in this manuscript:

ANCOVA	analysis of covariance
BMI	body mass index
CI	confidence intervals
CRP	C-reactive protein
DNA	deoxyribonucleic acid
ETS	E26 transformation-specific
HbA1c	hemoglobin A1C (glycated hemoglobin)
HDL	high-density lipoprotein
hTERT	human telomerase reverse transcriptase
HWE	Hardy-Weinberg Equilibrium
IQR	interquartile range
LDL	low-density lipoprotein
MAF	minor allele frequencies
mtDNA	mitochondrial DNA
MW	Mann-Whitney U
non-HDL	non-high-density lipoprotein
OR	odd ratio
PCR	polymerase chain reaction
RNA	ribonucleic acid
ROS	reactive oxygen species
RTL	leukocyte relative telomere length
SNPs	single-nucleotide polymorphisms
T1DM	type 1 diabetes mellitus
T2DM	type 2 diabetes mellitus
TC	total cholesterol
TCF	T-cell factor
TERC	telomerase RNA component
TG	triglycerides
WC	waist circumference
