# Supplementary material for: Telomere-Independent Associations of hTERT Promoter Polymorphisms with Metabolic Phenotypes and Diabetic Complications
Source: Int J Mol Sci. 2026 Oct 19;27(20):9292. doi: 10.3390/ijms27209292 (PMC13705372; doi:10.3390/ijms27209292)
Supplement: Supplementary file 1 [file ijms-27-09292-s001.zip › ijms-4604775-supplementary.pdf]

Table S1. False Discovery Rate (FDR)-adjusted sensitivity analysis (Benjamini-Hochberg) for candidate polymorphism associations (exploratory secondary outcomes).

Cohort	Polymorphism / Genetic Model	Clinical / Metabolic Parameter	Raw *p*-value	FDR-adjusted *p*-value
T1DM	rs7712562 Dominant	WC [cm]	0.016	1.000
T2DM	rs2735940 Recessive	TC [mg/dl]	0.030	1.000
T2DM	rs2735940 Recessive	LDL [mg/dl]	0.038	1.000
T2DM	rs2735940 Recessive	Non-HDL [mg/dl]	0.048	1.000
T2DM	rs2853669 Recessive	Diabetic neuropathy	0.048	1.000

p-values were adjusted for multiple testing using the Benjamini-Hochberg False Discovery Rate (FDR) procedure. The total number of exploratory secondary tests included in the family-wise adjustment was m = 261. TC – total cholesterol, LDL – low-density lipoprotein, Non HDL – non-high-density lipoprotein, WC – waist circumference.
